# Phylogeny and evolutionary history of *Leymus *(Triticeae; Poaceae) based on a single-copy nuclear gene encoding plastid acetyl-CoA carboxylase

**DOI:** 10.1186/1471-2148-9-247

**Published:** 2009-10-08

**Authors:** Xing Fan, Li-Na Sha, Rui-Wu Yang, Hai-Qin Zhang, Hou-Yang Kang, Cun-Bang Ding, Li Zhang, You-Liang Zheng, Yong-Hong Zhou

**Affiliations:** 1 Triticeae Research Institute, Sichuan Agricultural University, Wenjiang 611130, Sichuan, PR China; 2 Key Laboratory of Crop Genetic Resources and Improvement, Ministry of Education, Sichuan Agricultural University, Yaan 625014, Sichuan, PR China; 3 Department of Biology and Science, Sichuan Agricultural University, Yaan 625014, Sichuan, PR China

## Abstract

**Background:**

Single- and low- copy genes are less likely subject to concerted evolution, thus making themselves ideal tools for studying the origin and evolution of polyploid taxa. *Leymus *is a polyploid genus with a diverse array of morphology, ecology and distribution in Triticeae. The genomic constitution of *Leymus *was assigned as NsXm, where Ns was presumed to be originated from *Psathyrostachys*, while Xm represented a genome of unknown origin. In addition, little is known about the evolutionary history of *Leymus*. Here, we investigate the phylogenetic relationship, genome donor, and evolutionary history of *Leymus *based on a single-copy nuclear *Acc1 *gene.

**Results:**

Two homoeologues of the *Acc1 *gene were isolated from nearly all the sampled *Leymus *species using allele-specific primer and were analyzed with those from 35 diploid taxa representing 18 basic genomes in Triticeae. Sequence diversity patterns and genealogical analysis suggested that (1) *Leymus *is closely related to *Psathyrostachys*, *Agropyron*, and *Eremopyrum*; (2) *Psathyrostachys juncea *is an ancestral Ns-genome donor of *Leymus *species; (3) the Xm genome in *Leymus *may be originated from an ancestral lineage of *Agropyron *and *Eremopyrum triticeum*; (4) the *Acc1 *sequences of *Leymus *species from the Qinghai-Tibetan plateau are evolutionarily distinct; (5) North America *Leymus *species might originate from colonization via the Bering land bridge; (6) *Leymus *originated about 11-12MYA in Eurasia, and adaptive radiation might have occurred in *Leymus *during the period of 3.7-4.3 MYA and 1.7-2.1 MYA.

**Conclusion:**

*Leymus *species have allopolyploid origin. It is hypothesized that the adaptive radiation of *Leymus *species might have been triggered by the recent upliftings of the Qinghai-Tibetan plateau and subsequent climatic oscillations. Adaptive radiation may have promoted the rapid speciation, as well as the fixation of unique morphological characters in *Leymus*. Our results shed new light on our understanding of the origin of Xm genome, the polyploidization events and evolutionary history of *Leymus *that could account for the rich diversity and ecological adaptation of *Leymus *species.

## Background

*Leymus *Hochst., a polyploid perennial genus in the wheat tribe (Poaceae: Triticeae), includes about 30 species that distribute in a wide range of ecological habitats over the temperate and subtropical and tropic alpine regions [1-3]. The natural habitats of *Leymus *species range from coastal to inland areas, including saline or alkaline lands, dry or semi-dry areas, as well as shady and moist forests. Morphologically, *Leymus *species exhibit large variation with absent (*L. akmolinensis*) to strong rhizomes (*L. racemosus*), single (*L. ambiguus*) to multiple spikelets (*L. cinereus*) per node, erectly involute (*L. paboanus*) to loosely flat (*L. multicaulis*) leaf, and subulate (*L. innovatus*) to lanceolate (*L. arenarius*) to absent (*L. duthiei*) glumes [1,3-5].

The sectional delimitation of *Leymus *has been proposed by different scholars (See Table S1, Additional File 1). According to morphological characteristics, Tzvelev [4] and Löve [1] divided *Leymus *into four sections: sect. *Leymus*, sect. *Anisopyrum*, sect. *Aphanoneuron*, and sect. *Malacurus*. Barkworth and Atkins [5] suggested that the North American species of *Leymus *cannot be separated into sect. *Anisopyrum *and sect. *Aphanoneuron *and recognized *Leymus *as sect. *Leymus *and sect. *Anisopyrum*. Zhi and Teng [6] divided Chinese *Leymus *into three sections: sect. *Racemosus*, sect. *Leymus*, and sect. *Anisopyrum*, and suggested that central Asia might be the centre of diversity of the genus. Based on ecological habitats, Yen and Yang [3] defined three ecological sections of *Leymus*: sect. *Arenarius*, sect. *Pratensus*, and sect. *Silvicolus*. While these studies add to our understanding of subdivision of *Leymus*, phylogenetic relationships among its species remain unclear. Moreover, little is known about the evolutionary history of *Leymus*.

Cytologically, five ploidy levels were recognized in *Leymus *species: tetraploid (2n = 4x = 28), hexaploid (2n = 6x = 42), octoploid (2n = 8x = 56), decaploid (2n = 10x = 70) and dodecaploid (2n = 12x = 84) [1,5]. *Leymus *has its origin through a typical polyploidization process, which might originate from allopolyploidy for ancestral entity and then continuous autopolyploidy for higher polyploids [1,2]. All the *Leymus *species have two basic genomes, Ns and Xm [7]. Previous studies based on morphology [1], cytogenetics [8], DNA hybridization patterns [9], and DNA sequences (nrITS, *trnL-F*) [10,11] have revealed that the Ns genome of *Leymus *was originated from the genus *Psathyrostachys*. Despite decades of intensive efforts, there are still uncertainties regarding the origin of the Xm genome of *Leymus*. Based on morphological characteristics, the Xm genome was presumed to be the St genome of *Pseudoroegneria *[12] or the E^b ^genome of *Thinopyrum bessarabicum *[1]. Cytogenetic analysis suggested that it was the E^e ^genome of *Lophopyrum elongatum *[13]. However, cytogenetic and DNA hybridization analysis excluded the E^b ^and E^e ^genomes from the *Leymus *species [8,9,14]. Zhang and Dvorak [9] and Bödvarsdóttir and Anamthawat-Jónsson [15] advocated that tetraploid *Leymus *species were segmental autotetraploids with the genomes Ns_1_Ns_1_Ns_2_Ns_2_, derived from two distinct *Psathyrostachys *species. Wang et al. [7] designated the genomes of *Leymus *species as NsXm until the source of Xm is identified.

Polyploidy, including autopolyploidy and allopolyploidy, is a simple speciation process. The success of polyploids is often attributed to their genomic changes including genomic stability, chromosomal rearrangement, genome size and differential gene expression, which allows polyploids to adapt to new ecological niches or to be competitively superior to the parental diploids [16]. A better understanding of the process of polyploidization and evolutionary history of polyploids is of widespread evolutionary interest [16]. Phylogenetic analyses of chloroplast and nuclear DNA data have increased the possibility to recognize polyploidy and hybridization in plants [16-18]. However, the often relatively low variation in chloroplast DNA at the intraspecific level have limited the precision with which polyploidy can be identified [17,19]. Single- and low- copy nuclear genes are less likely subject to concerted evolution, thus making themselves ideal tools for verifying the existence of suspected polyploids [20], identifying genome donors [18,21], demonstrating multiple polyploid origins [19], clarifying hybridization events or introgression [18], and examining gene evolution in polyploids [22]. Plastid acetyl-CoA carboxylase (ACCase) catalyzes the first step in *de novo *fatty acid biosynthesis. Southern hybridization and chromosome mapping indicate that a single copy of the *Acc1 *gene is present in each of the group 2 homoeologous chromosomes in hexaploid wheat [23]. The *Acc1 *gene has been successfully used to study the phylogenetic relationships and evolutionary history of *Triticum/Aegilops *complex [24], and the evolution of *Panicum virgatum *L. [25].

In this study, we sequenced and analyzed the single-copy nuclear *Acc1 *gene for 29 *Leymus *polyploids and 35 diploid taxa representing 18 basic genomes in Triticeae. The objectives were (1) to elucidate the phylogenetic relationships of the *Leymus *species; (2) to demonstrate the evolution of *Leymus*; (3) to explore the origin of the unknown Xm genome in *Leymus*; (4) to estimate the divergence times for nodes within *Leymus *to document biogeographic diversification history of the genus.

## Methods

### Taxon sampling

Twenty-eight species and one variety of *Leymus *were included in this study. They were analyzed together with 35 diploid taxa representing 18 basic genomes in the tribe Triticeae. Sample information and GenBank accession data were listed in Table S2 (See Additional file 2). The seed materials of *Leymus *with PI and W6 numbers were kindly provided by American National Plant Germplasm System (Pullman, Washington, USA), and *Leymus duthiei *var. *longearistata *was kindly provided by Dr. S. Sakamoto (Kyoto University, Japan). The seed materials of *Leymus *with ZY and Y numbers were collected from the field by the authors of this paper. The plants and voucher specimens of the *Leymus *species are deposited at Herbarium of Triticeae Research Institute, Sichuan Agricultural University, China (SAUTI).

### DNA Amplification, homoeologous sequence isolate, and sequencing

DNA extraction followed a standard CTAB protocol [26]. The *Acc1 *gene was amplified with the *Acc1*-specific primers AccF1 (5'-CCCAATATTTATCATGAGACT TGCA-3') and AccF2 (5'-CAACATTTGAATGAAThCTCCACG-3'), and PCR was conducted under cycling conditions reported previously [24]. To decrease the chance of PCR drift and PCR selection, a 75 μl reaction mixture was separated into five reactions, and PCR products were pooled together after amplification. The *Acc1 *sequence amplification was carried out in a 15 μl reaction mixture consisted of 0.3 U of high-fidelity Ex *Taq *DNA polymerase (TaKaRa Biotechnology Co. Ltd., Dalian, China), 1× reaction buffer, 1.5 mM MgCl_2_, 1.2 mM of each dNTP, 0.5 μM of each primer. PCR products were cloned into the pMD18-T vector (TaKaRa) following the manufacture's instruction.

Cloning of PCR amplicons from single-copy nuclear genes from allopolyploid species will isolate homoeologous sequences from each nuclear genome [27]. Given that *Leymus *species contain the Ns and Xm genomes, PCR reactions will produce a heterogeneous mix of *Acc1 *sequences from each *Leymus *polyploid. To separate the homoeologous sequence of *Acc1 *gene from each accession, we performed the following process. Firstly, approximately 20 positive clones from each accession were screened by direct PCR using primer AccF1 coupled with M13R (on the side of the cloning site in the plasmid). Secondly, an Ns-type *Acc1*-specific primer, AccFn (5'-GAACCTGTGCTCATATGGTATATTA-3'), was designed and used together with the reverse primer AccF2 to screen the Ns-type *Acc1 *sequences from above 20 positive clones with *Acc1 *inserts. Thirdly, the rest of positive clones including the Xm-type *Acc1 *sequences were obtained. Finally, to determine whether the Xm-type of *Acc1 *sequences was the result of a biologically relevant recombination, an Xm-type *Acc1*-specific primer, AccFx (5'-ACTGCAGGTATGTTCTTTT-3'), was designed and used together with the primer AccF2 to amplify the Xm-type *Acc1 *sequences. The cloned PCR products were sequenced in both directions by TaKaRa Biotechnology Co. Ltd. (Dalian, China). All the sequences from *Leymus *species were determined at least 5 independent Ns-type clones and 5 independent Xm-type clones.

### Alignments and test for substitution saturation

Multiple sequences were aligned using ClustalX [28], with default options (gap opening/gap extension: 15/6.66), and the alignments were refined manually in an effort to maximize the positional homology. To reduce the size of the matrixes and the possible impact of PCR artifacts, unique substitutions in single clones were ignored and several identical sequences were represented by a single original sequence in alignments. In the initial phylogenetic analysis, the number of sequences used for alignment was reduced by keeping only one sequence if more sequences of the same accession formed a monophyletic group. Substitution saturation in the alignments was estimated by plotting pairwise rates of transitions and transversions against sequence divergence calculated under TN93 model using the software DAMBE version 5.0.7 [29].

### Nucleotide diversity estimate

To assess the divergence and genetic relationships between polyploids and its diploid progenitor, nucleotide diversity was estimated by Tajima's *π *[30], Watterson's *θ *[31], the number of fixed differences (S_F_) and the numbers of shared polymorphisms (S_S_). Tajima's *π *quantifies the mean percentage of nucleotide differences among all pairwise comparisons for a set of sequences, while Watterson's *θ *is simply an index of the number of segregating (polymorphic) sites. A fixed difference refers to the nucleotide site where all sampled sequences from one taxon are different from all sequences from another taxon, whereas a shared polymorphism occurs when two taxa have the same two bases segregating at the same site [32]. Closely related taxa are expected to harbor a relative higher level of shared polymorphisms because the divergence event has not lasted long enough to erase all ancestral polymorphisms. Tests of neutrality including Tajima's and Fu and Li's *D *statistic were performed as described by Tajima [30], and Fu and Li [33]. Significance of *D*-values was estimated with the simulated distribution of random samples (1000 steps) using a coalescence algorithm assuming neutrality and population equilibrium [34]. These parameters were calculated with DnaSP 4.10.9 [35] and ProSeq 2.0 [36].

We also examined demographic history in sampled *Leymus *lineages using mismatch distributions [37] and Fu's *F*s statistics [38] in the program DnaSP 4.10.9 [35]. Mismatch distributions are frequency distributions of observed nucleotide pairwise differences, and allow to infer whether lineages have undergone sudden or stepwise demographic expansion during historical periods [37]. A unimodal mismatch distribution indicates a recent expansion, a multimodal (including bimodal) mismatch distribution indicates diminishing population sizes or structured size. The Fu's *F*s statistic is very sensitive to demographic expansion [38]. Significance of *F*s-values was calculated with the simulated distribution of random samples (1000 steps) [34].

### Phylogenetic analysis

Three data matrixes, including exon + intron data, intron data and exon data, were used separately to carry out phylogenetic analyses. Phylogenetic analyses were conducted using maximum likelihood (ML) and Bayesian inference (BI). ML analysis was performed using PAUP*4.0b10 (Swofford D L, Sinauer Associates, ). *Bromus inermis *Leyss. was used as the outgroup. The evolutionary model used for the three different data matrixes was determined using ModelTest v3.0 with Akaike information criterion (AIC) [39]. The optimal models identified were GTR + G + I for the exon + intron data, GTR + G for the intron data, and TIM + G + I for the exon data. ML heuristic searches were performed with 100 random addition sequence replications and TBR branch swapping algorithm. The robustness of the trees was estimated by bootstrap support (BS) [40]. ML bootstrapping was performed with 250 replicates, each with three replicates of stepwise random taxon addition, using the same model and parameters. BS-value less than 50% was not included in figures.

Bayesian inference (BI) analysis was performed using MrBayes v3.0 [41]. BI analyses of the exon + intron and intron data matrixes were carried out under the same evolutionary model as ML analysis. For the exon data, because the TIM + G + I model is not implemented in MrBayes v3.0, we chose the closely related GTR + G + I model instead. Four MCMC (Markov Chain Monte Carlo) chains (one cold and three heated), applying MrBayes default heating values (*t *= 0.2), were run for 3,800,000 generations for the exon + intron data, 2,000,000 generations for the intron data, and 9,000,000 generations for the exon data, each sampling every 100 generations. The first 9500, 4200, and 23000 trees were stationary discarded as "brun-in" for the exon + intron, intron, and exon, respectively. The program Tracer v1.4 [42] was used to examine the log likelihoods, ensuring that they were in the stationary "fury caterpillar" phase. The remaining trees were used to construct the 50%-majority rule consensus trees. Two independent runs were performed to check whether convergence on the same posterior distribution was reached. The statistical confidence in nodes was evaluated by posterior probabilities (PP). PP-value less than 90% was not included in figures.

### Network analysis

Taking the potential for reticulation in closely related lineages into consideration, phylogenetic network reconstruction method was used to study relationships between ancestral and derived haplotypes. The median-joining (MJ) network method was performed for this study because of robustness compared to other network methods in simulation studies using known gene genealogies [43]. MJ network has been successfully used to reveal specific progenitor-descendant relationships of polyploidy *Triticum *within Triticeae [44].

MJ network analysis was generated by the Network 4.1.1.2 program (Fluxus Technology Ltd, Clare, Suffolk, UK). As the program infers median-joining networks from non-recombining DNA [45], the test of recombination was performed using the GARD recombination-detection method within the HyPhy package [46]. Building upon this test, the exon data was used to generate MJ network because of the absence of recombination signal in alignment (Log Likelihood = -1911.85; AIC = 4061.69), while the exon + intron and intron data were not used to reconstructed MJ network due to potential recombination signal.

### Divergence dating

The hypothesis of rate constancy was evaluated with a likelihood ratio test comparing the likelihood scores from the unconstrained and clock-constrained analyses. The molecular clock was rejected because constrained and unconstrained analyses differed significantly (χ^2 ^= 205.94, df = 88, *P *< 0.0001). Therefore, divergence times with 95% confidence intervals (C.I.) were estimated using Bayesian relaxed molecular clock method, implemented in BEAST v1.4.6 [47]. The lack of fossils for Triticeae precluded a direct calibration of tree topologies. Instead, node dating of the intron data was estimated on the basis of the intron region of the *Acc1 *gene clock of 0.0036 substitutions per site per MY (million year) [48]. Two relaxed clock models, including uncorrelated lognormal and exponential branch length distribution models, were used to perform node dating. The final dating from the two models was compared. MCMC searches were run for 10,000,000 generations under GTR + G model (with the associated parameters specified by ModelTest as the priors), with the first 2,000,000 discarded as burn-in. The searches achieved adequate mixing as assessed by the high ESS values for all parameters, plateaus for divergence time estimates over generations after burn-in. For all analyses two independent runs were performed, the log files were combined to check for convergence on the same distribution and to ensure adequate sample sizes, and viewed using Tracer v.1.4 [42].

## Results

### Screening of homoeologous Acc1 sequences in Leymus

About 20 forward (5'-3' direction) clones were screened from a plate of clones with putative *Acc1 *inserts by the primer AccF1 and M13R. The Ns-type *Acc1 *sequences were screened from above 20 forward clones by the primer AccFn and AccF2, and the rest of clones were the putative Xm-type *Acc1 *sequences. The primer AccFx and AccF2 were further used to verify the presence of the Xm-type *Acc1 *sequences. Consequently, two distinct types of *Acc1 *sequences (Ns- and Xm-type) were obtained from 24 *Leymus *species. Only one type of *Acc1 *sequence was detected from *Leymus multicaulis *(Ns-type), *L. coreanus *(Ns-type), *L. ramosus *(Xm-type), *L. duthiei *var. *longearistata *(Xm-type) and *L. akmolinensis *(Xm-type). For these five taxa, alternative genome type might be obtained by more screens with extensive positive clones if they were allopolyploidy origin. Another possibility is that one type has been pseudogenized so that they are undetectable by our primers. In addition, we also cannot rule out the possibility of autopolyploidy of all (or partial) five taxa with only one type of *Acc1 *sequence.

### Acc1 sequence analyses

**Figure 1:**
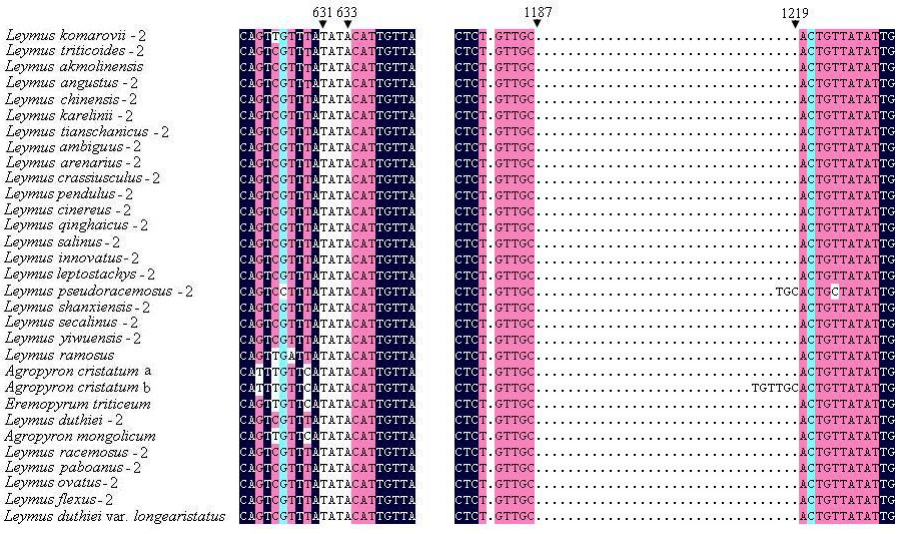
**Partial alignment of the amplified sequences of *Acc1 *gene from *Leymus *and its affinitive species**. A 4-bp TATA insertion at position 631-633 and a 33-bp insertion at position 1187-1219 are labeled. This figure shows the upper quartile, for the full image please see Additional file 3.

The DNA sequence of the *Acc1 *gene includes 8 exons and 7 introns, which was in agreement with previous studies [24]. The sequence comparison from all the species studied here showed that the DNA sequence ranged in length from 1391 bp to 1470 bp, and the DNA sequences in most accessions were ~ 1440 bp in size. The aligned sequence length of the exon + intron, exon and intron data sets were 1549 bp, 696 bp and 857 bp, respectively. The alignment of the exon sequence was unambiguous without gaps. Many gaps, which resulted from indels (insertion/deletion) within intron regions, were found in the alignment of the exon + intron and intron data sets. In particular, apart from the single nucleotide acid substitution and deletion, the Xm-type sequences of *Leymus *species and the sequences from *Agropyron *species and *Eremopyrum triticeum *had a 4-bp TATA insertion at position 631-633 in the intron region compared to the remaining sequences (See Figure 1 and Additional file 3). A 33-bp insertion was detected for the Ns-type sequences at position 1187-1219 in the intron region from 11 *Leymus *species (*L. flexus*, *L. qinghaicus*, *L. leptostachys*, *L. pseudoracemosus*, *L. ovatus*, *L. pendulus*, *L. secalinus*, *L. crassiusculus*, *L. duthiei*, *L. shanxiensis *and *L. yiwuensis*) (See Additional file 3).

Features of the exon + intron, exon and intron data matrixes were listed in Table 1. As expected, due to strong functional constraint, the levels of nucleotide variation in exon region (150 variable characters, 9 transition mutations, and 3 transversion mutations) were lower than those in intron region (407 variable characters, 25 transition mutations, and 19 transversion mutations). The ratio of parsimony-informative characters and variable characters was 0.521 (intron), 0.496 (exon + intron), and 0.427 (exon), respectively. Plotting pairwise rates of transitions and transversions against sequence divergence calculated under TN93 model revealed a nearly linear regression, indicating that the three sequence data sets showed no saturation effects.

**Table 1:** Features of the three matched data matrix

	**Variable characters**	**Conserved characters**	**Informative characters**	**ii**	**si**	**sv**
Exon	150	546	64	684	9	3
Intron	407	410	212	690	25	19
Exon+Intron	560	953	278	1374	34	22

### Phylogenetic analyses

To reveal the putative genome donors of *Leymus*, the *Acc1 *gene sequences of all the polyploid species were included in the phylogenetic analyses, together with 35 diploid taxa representing 18 genomes in Triticeae (See Table S2, Additional file 2). At least 10 positive clones (including 5 Ns-type and 5 Xm-type clones) were sequenced. In cases multiple identical sequences resulted from cloned PCR products of one accession, only one sequence was included in the data set. Consequently, 89 unique sequences were obtained and used for the phylogenetic and network analysis.

**Figure 2:**
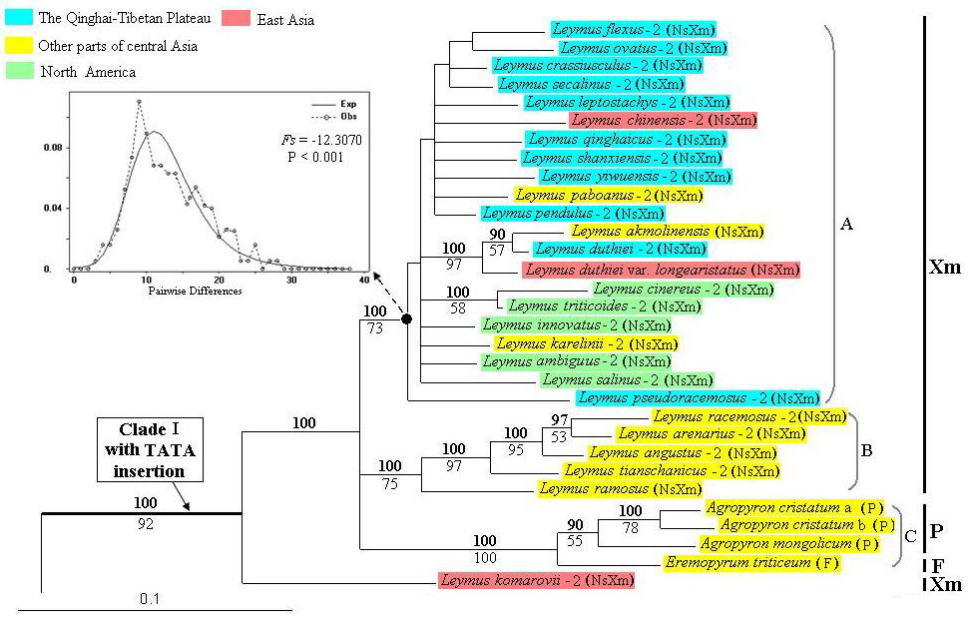
**Maximum-likelihood tree (-Lnlikelihood = 8827.1373, base frequencies A: 0.2432, C: 0.1830, G: 0.2152, T: 0.3586, shape = 0.8022, pinvar = 0.1926) inferred form the exon + intron sequences of the *Acc1 *gene of *Leymus *and its affinitive species, under GTR + G + I model**. Numbers with bold above nodes are Bayesian posterior probability values ≥ 90% numbers below nodes are bootstrap values ≥ 50%. The numbers after species names refer to the distinct homoeologous of *Acc1 *gene. The capital letters in bracket indicate the genome type of the species. Different color labeled the geographic information of *Leymus *species and its putative donor. Boxed subset provides mismatch distribution and *F*s statistic for the *Acc1 *sequence of taxa at the node marked with black dot. The letter a and b represent two different accessions of *Agropyron cristatum*. This figure shows the upper quartile, for the full image please see Additional file 4.

Three data sets (exon + intron, intron, and exon) were used separately to perform phylogenetic analyses (ML and BI). ML analysis of the exon + intron data yielded a single phylogenetic tree (-Lnlikelihood = 8827.1373), with the following estimated ML parameters: the assumed nucleotide frequencies A: 0.2432, C: 0.1830, G: 0.2152, T: 0.3586, the proportion of invariable sites = 0.1926, gamma shape parameter = 0.8022. ML and Bayesian analyses of the exon + intron data recovered the same topology. The tree illustrated in Figure 2 was the ML tree of posterior probabilities (PP) above and bootstrap support (BS) below branches.

The phylogenetic tree showed that the Ns-type and Xm-type sequences from *Leymus *species were split into two well supported clades, clade I and clade II (See Figure 2 and Additional file 4). The clade I included the Xm-type sequences of *Leymus *and the sequences from *Ag. cristatum*, *Ag. mongolicum*, and *Er. triticeum*. Three subclades (A, B, and C subclade) with high statistical support were recognized in this clade. Subclade A included all the *Leymus *species from North America and the Qinghai-Tibetan Plateau, three *Leymus *species from other parts of central Asia and one *Leymus *taxa from East Asia (100% PP and 73% BS). Subclade B consisted of four *Leymus *species from Xiangjiang of China and one *Leymus *species from Kazakhstan (100% PP and 75% BS). Subclade C contained three *Agropyron *accessions and *Er. triticeum *(100% PP and 100% BS). *Leymus komarovii *was placed outside Subclade A, B, and C. The clade II contained the Ns-type sequences of *Leymus *and the sequences from *Psathyrostachys*. Two subclades (D and E subclade) with high statistical support were recognized within the clade II. Subclade D included four *Psathyrostachys *species and all the *Leymus *species except for those from the Qinghai-Tibetan Plateau (100% PP and 71% BS). Subclade E contained the *Leymus *species from the Qinghai-Tibetan Plateau (100% PP and 80% BS). It was worth mentioning that the sequence in subclade E had a 33-bp insertion at position 1187-1219 (See Additional file 4). Although subclade A, D and E were supported by high bootstrap values and posterior probabilities, the phylogenetic relationships among species within these subclades were considered unresolved due to a high number of zero-length branches.

**Figure 3:**
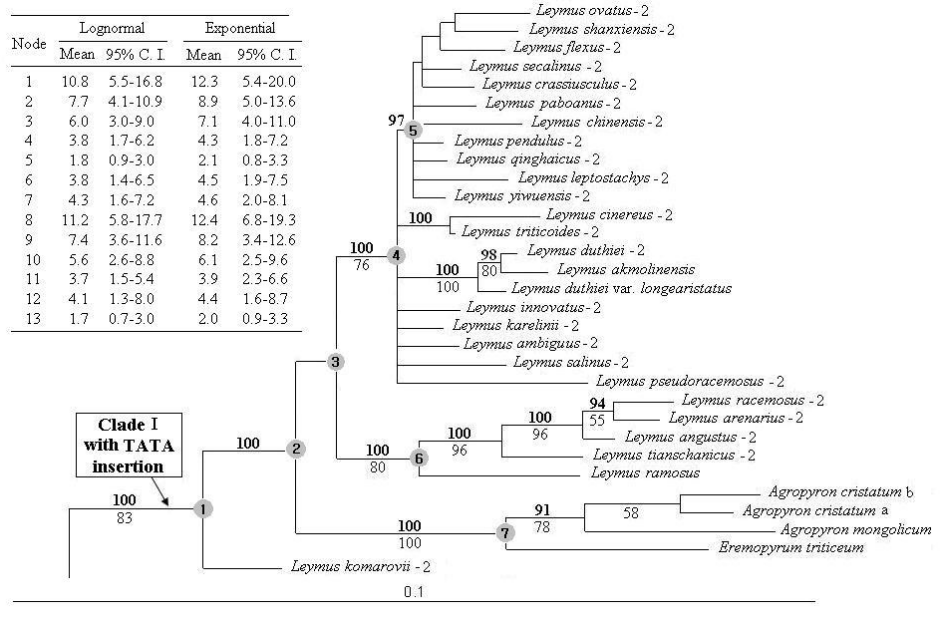
**Fifty-percent majority-rule Bayesian tree inferred form the intron sequences of nuclear *Acc1 *gene of *Leymus *and its affinitive species, under GTR + G model**. Numbers with bold above nodes are Bayesian posterior probability values ≥ 90% numbers below nodes are bootstrap values ≥50%. The numbers after species names refer to the distinct homoeologous of *Acc1 *gene. The table provides the estimated divergence dates for nodes labeled 1-13. The letter a and b represent two different accessions of *Agropyron cristatum*. This figure shows the upper quartile, for the full image please see Additional file 5.

ML analysis of the intron data yielded a single phylogenetic tree (-Lnlikelihood = 5903.3227), with the assumed nucleotide frequencies A: 0.2250, C: 0.1823, G: 0.1755, T: 0.4172, gamma shape parameter = 0.9703. ML and Bayesian analyses of intron data generated the same topology. The tree illustrated in Figure 3 was the BI tree of posterior probabilities (PP) above and bootstrap support (BS) below branches. This tree was highly congruent with the tree inferred from the exon + intron data except for some nodes presenting different statistical support (See Figure 3 and Additional file 5). Those polytomies observed in the tree inferred from the exon + intron data (within subclade A, D, and E) was not resolved with the use of intron data.

**Figure 4:**
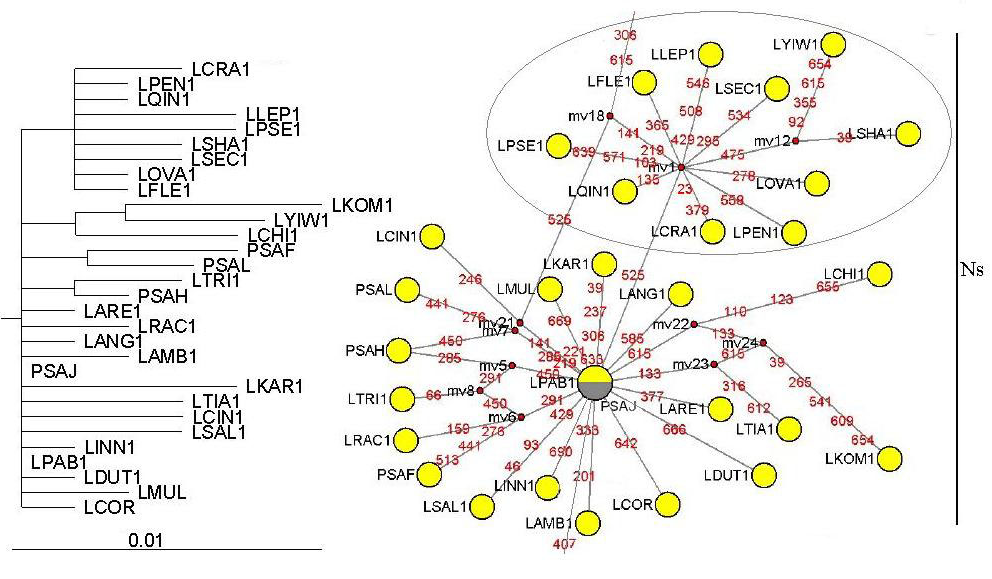
**Maximum-likelihood tree (left) and Median-joining networks (right) based on exon haplotype of *Leymus *and its affinitive species**. Maximum-likelihood tree (-Lnlikelihood = 5903.3227, base frequencies A: 0.2630, C: 0.1880, G: 0.2688, T: 0.2802, shape = 0.9988, pinvar = 0.4451) was generated under TIM + G + I model. Numbers with bold above nodes are Bayesian posterior probability values ≥ 90%; numbers below nodes are bootstrap values ≥50%. Haplotypes in network are represented by circles. Numbers along network branches indicate the position of mutation between nodes. Abbreviations of species names are listed in table 1. The numbers after species names refer to the distinct homoeologous of *Acc1 *gene. The letter a and b represent two different accessions of *Agropyron cristatum*. This figure shows the middle quartile, for the full image please see Additional file 6.

ML analysis of the exon data resulted in a single phylogenetic tree (-Lnlikelihood = 1877.7337), with the assumed nucleotide frequencies A: 0.2630, C: 0.1880, G: 0.2688, T: 0.2802, the proportion of invariable sites = 0.4451, gamma shape parameter = 0.9988. The ML trees showed that the Ns-type and Xm-type sequences from *Leymus *species were split into two clades but with weak bootstrap support (See Figure 4 and Additional file 6). BI and ML analysis of the exon data recovered the similar topology. Although basic topology based on the exon data was congruent with the tree inferred from the exon + intron and intron data, many low resolution branches were showed.

### Network analyses

To get better insights into the number of haplotypes of the *Acc1 *sequence and their relatedness, the network reconstruction methods was employed. The exon data was used to generate MJ network because no recombination signal was detected in its alignment. Each circular network node represents a single sequence haplotype, with node size being proportional to number of isolates with that haplotype. Mv (median vectors representing missing intermediates) shows unsampled nodes inferred by MJ network analysis, and the number along the branches shows the position of mutations. Network loops represent either true reticulation events or alternative genealogies in closely related lineages.

Eighty-three haplotypes were derived from 89 sequences. MJ network revealed higher levels of haplotype diversity of exon region (See Figure 4 and Additional file 6). Two distinct types of haplotypes (Ns- and Xm-type) of *Leymus *species were recognized. The Ns haplotypes were four mutational steps (at position 267, 268, 306, and 615) away from the Xm haplotypes. In the Ns-type haplotype, *Psa. juncea *and *L. paboanus *were placed at central branching points, while the haplotypes from the Qinghai-Tibetan Plateau formed one distinct group. In the Xm-type haplotype, all the haplotypes from *Leymus *species formed one star-like radiation, and *L. crassiusculus *(LCRA2), *L. leptostachys *(LLEP2), *L. innovatus *(LINN2) and *L. ambiguus *(LAMB2) were placed at central branching points. The Xm-type haplotype was closely related to those from *Ag. cristatum*, *Ag. mongolicum *and *Er. triticeum*.

### Nucleotide diversity between Leymus and its diploid progenitor

The greatest pairwise distance among the Ns-type sequences of *Leymus *ranged from 0.210 to 3.377%, while the greatest pairwise distance among the Xm-type sequences of *Leymus *ranged from 0.208 to 2.401%. The levels of divergence between the Xm-type sequences of *Leymus *and the sequences from *Agropyron *species and *Eremopyrum triticeum *was 4.477%. Sequence divergence between the Ns-type sequences of *Leymus *and Ns sequences of *Psathyrostachys *was 2.960%. The levels of divergence between Ns-type and Xm-type sequences within *Leymus *was 6.149%. These results indicated that the Ns- and Xm-type sequences in *Leymus *species were correspond to the two homoeologous *Acc1 *sequences.

Estimates of nucleotide polymorphism, π and θ, were shown separately for the Ns genome of *Leymus *and *Psathyrostachys*, and for Xm genome of *Leymus *(Table 2). The number of polymorphic sites in the Ns genome of *Leymus *was higher than that in the Ns genome of diploid *Psathyrostachys*. The estimates of Tajima's and Fu and Li's D statistic for *Acc1 *gene on the Ns genome of *Leymus *were -1.4676 (P < 0.05) and -2.5042 (P < 0.05), respectively, while Tajima's D estimate on the Ns genome of diploid species was 0.65817 (P > 0.10).

**Table 2:** Estimates of nucleotide diversity and test statistics at *Acc1 *locus in *Leymus *Ns and Xm genome and its putative diploid genome donor

	***n***	***s***	***π***	***θ*_*w*_**	**Fu & Li's D**	**Tajima's D**
*Leymus*						
Ns	1429	153	0.0176	0.0281	-2.5042 (P < 0.05)	-1.4676 (P < 0.05)
Xm	1424	145	0.0123	0.0262	-2.9884 (P < 0.05)	-2.0627 (P < 0.05)
*Psathyrostachys*						
Ns	1433	27	0.0109	0.0103	-0.0425 (P > 0.10)	0.6582 (P > 0.10)

The *n *is the number of the sites (excluding sites with gaps/missing data), *s *is the number of segregating sites, *π *is the average pairwise diversity, and *θ*_*w *_is the diversity based on the number of segregating sites.

Thirteen shared polymorphisms and no fixed difference were observed at *Acc1 *locus between the Ns-type sequence of *Leymus *and that of *Psathyrostachys*, while equal numbers of shared polymorphisms and fixed differences (Ss = 8, S_F _= 8) were observed between the Xm-type sequence of *Leymus *and the sequences from *Agropyron *species and *Er. triticeum*. This indicated that *Leymus *are closer to *Psathyrostachys *than to *Agropyron *and *Eremopyrum*.

### Mismatch distribution and Fs statistic

Mismatch distribution and Fu's *F*s statistic were used to infer whether *Leymus *lineages within those terminal clades with low resolution branches have undergone sudden or stepwise expansion during historical periods. Observed pairwise differences (mismatch distributions) between *Acc1 *sequences within subclade A, D, and E were all unimodal (See Figure 2 and Additional file 4). Fu's *F*s statistic of *Acc1 *sequences within subclade A, D, and E were -12.3070 (P < 0.001), -7.0359 (P < 0.001), and -3.6640 (P < 0.001), respectively. These results indicate that *Leymus *lineages species had undergone expansion during its evolutionary history.

### Node dating

Thirteen nodes were dated (nodes 1-13 in Figure 3 and Additional file 5) using the intron region of the *Acc1 *gene molecular clock of 0.0036 substitutions per site per MY. Two age estimates with 95% confidence intervals based on two different relaxed clock models were also presented in Figure 3 (See also Additional file 5). Under a lognormal relaxed clock, the coefficient of rate variation was estimated to be 0.752 (95% C.I., 0474-1.007), indicating that the intron data was not strictly clock-like and relaxed clock was appropriate. The age of divergence represent an interval depending on the model of branch length distribution (each with 95% C. I.). Time calibration analysis demonstrated that the age for the divergence of *Leymus *was dated to 10.8-12.4 MYA (node 1 and 8). Two extensive radiations of *Leymus *were dated to 3.7-4.3 MYA (node 4 and 11) and 1.7-2.0 MYA (node 5 and 13), respectively.

## Discussion

### Phylogenetic relationships in Leymus species

Two types of *Acc1 *sequences, Ns- and Xm-type, were obtained from nearly all the polyploidy *Leymus *species in the present study. This allows phylogenetic relationships among *Leymus *species to be elucidated on the basis of orthologous comparison.

ITS data presented by Liu et al. [10] and Sha et al. [11] showed that the *Leymus *species from North America were grouped together with some *Leymus *species from central Asia. By analyzing random amplified polymorphic DNA polymorphism (RAPD), Yang et al. [49] suggested that the *Leymus *species from North America were scattered in different subclades and grouped with those species from central Asia. The present *Acc1 *gene data showed that the *Leymus *species from North America also did not group together but placed scatteredly into one subclade (subclade A and D) including the *Leymus *species from central Asia and East Asia. This indicates that the *Leymus *species from North America is closely related to those from central Asia and East Asia.

Based on ITS sequence data, Sha et al. [11] reported that *L. racemosus*, *L. arenarius*, *L. ramosus *and *L. paboanus *were nested into one group, while *L. angustus*, *L. tiancshanicus*, and *L. karelinii *were in another group. Anamthawat-Jónsson and Bödvarsdóttir [50] suggested that the northern European *L. arenarius *was closely related to the central Eurasian *L. racemosus *from fluorescence in situ hybridization (FISH) evidence. In this study, the *Acc1 *gene data revealed a close relationship of Xm genomes among *L. racemosus*, *L. arenarius*, *L. angustus*, *L. tiancshanicus*, and *L. ramosus*, which was partly congruent with previous study.

In the *Acc1 *gene tree, the Ns-type sequences of eleven *Leymus *species from the Qinghai-Tibetan Plateau were clustered distinctly in one clade, and the Xm-type sequences of most *Leymus *species from the Qinghai-Tibetan Plateau were also grouped together. Exon analysis showed that the *Leymus *species from the Qinghai-Tibetan Plateau were radiated from a putative unsampled node. These results suggest that the Ns-type sequences of *Acc1 *gene of *Leymus *species from the Qinghai-Tibetan Plateau and other parts of central Asia, East Asia and North America were evolutionarily distinct.

*L. komarovii *was previously described as *Hystrix komarovii *[3]. Ellneskog-Staam et al. [51] advocated that *L. komarovii *most likely had one variation of StH genomes of *Elymus*. Yen and Yang [3] regarded *L. komarovii *as section *Silvicolus *of *Leymus *species (forest-dwelling) on the basis of ecological habitats. Morphylogically, *L. komarovii *is distinguished from other *Leymus *species by glume subulate, often reduced at top, and awn equaling lemma boby. Analysis of ITS sequences by Sha et al. [11] showed that *L. komarovii *was placed at the base of the clade including all sampled *Leymus *species. In this study, two distinct *Acc1 *sequences were obtained from *L. komarovii*. One sequence was sister to the sequences of *Psathyrostachys *species and the Ns genome sequence of *Leymus *species except for those from the Qinghai-Tibetean Plateau, while the other sequence was placed at the base of the clade I including all the Xm genome sequence of *Leymus *and P/F genome sequence. This result, in combination with morphological and ITS sequence data, suggested that *L. komarovii *has the NsXm genome of *Leymus *but with some variation.

### Origin of Ns genome in Leymus

Meiotic pairing data from interspecific and intergeneric hybrids [2,8] and molecular studies [5,11,52] had revealed that the Ns genome of *Leymus *(NsXm) was originated from *Psathyrostachys*. Wakeley and Hey [53] pointed out that closely related species are expected to harbor a relative higher level of shared polymorphisms than fixed differences. In this study, *Leymus *was grouped with *Psathyrostachys *species, and shared polymorphisms and fixed differences (Ss = 13, S_F _= 0) were observed. These results indicate that *Leymus *is closely related to *Psathyrostachys*. Combined with previous cytological and molecular studies, it can be concluded that the *Psathyrostachys *species served as the Ns genome donor during the polyploid speciation of the *Leymus *species.

The Ns genome is represented in all *Leymus *species. *Psathyrostachys *is the Ns haplome donor genus, which contains approximately eight diploids (NsNs) or tetraploids (NsNsNsNs) speices distributed in the Middle East, central Asia, and northern China [3]. Based on DNA hybridization and FISH analyses, Wang et al. [54] suggested that the Ns genome of *Leymus *species may be originated from *Psa. juncea *and *Psa. lanuginosa*, while *Psa. fragilis *and *Psa. huashanica *were unlikely to be the donor species of the Ns genome in *Leymus *species. The present MJ analysis showed that all the exon haplotyes of Ns-type form a star-like radiation, and *Psa. juncea *was placed at central branching points, indicating that *Psa. juncea *is likely ancestral Ns-genome donor of *Leymus *species. The *Acc1 *gene genealogical structure may be related to the geographic ranges of *Psathyrostachys *species. *Psa. juncea *is widely distributed in the former USSR, Mongolia, and northwestern part of China, while *Psa. fragilis*, *Psa. huashanica *and *Psa. lanuginosa *is restrictly distributed in some regions of central Asia.

Polyploids often originate many times from independent populations of their progenitor, especially when polyploids and their parental species coexist on a large geographic scale [55]. Wei et al. [56] reported that considerable genetic heterogeneity was exhibited in accessions of *Psa. juncea *that have different geographical origins. On the basis of our *Acc1 *sequences, nucleotide sequence diversity (*π*) of the Ns genome of *Leymus *was higher than in the Ns genome of diploid *Psathyrostachys*, indicating that the *Acc1 *sequence may evolve faster in the polyploid species than in the diploids. Considering a large-scale sympatric distribution between *Leymus *species and *Psa. juncea*, another possible explanation for greater diversity in the Ns genome of *Leymus *is that *Leymus *experienced recurrent hybridization with different populations of *Psa. juncea*. This possibility is further supported by tests of selection history at *Acc1 *locus. Significantly negative values of Tajima's and Fu and Li's D statistic for the *Acc1 *gene on *Leymus *Ns and Xm genome indicates that the observed number of rare variations exceeds the expected number in an equilibrium neutral model, which might suffer from a genetic bottleneck created by polyploidization. Whereas Tajima's D estimate for the *Acc1 *gene on diploid Ns genome was positive. These estimates are indicative of an excess of rare variants in *Leymus *that could be created by recurrent hybridization event or introgression of Ns-genome during polyploidization.

### Possible Origin of the Xm Genome in Leymus

The genomic constitution of *Leymus *was assigned as NsXm, where Xm represented a genome of unknown origin [7]. Three different genomes Ns [9,50], St [12] and J(E^b^) [1,2] have been suggested as Xm genome. Sun et al. [13] suggested that *Leymus secalinus *might have 3-4 chromosomes from the E^e ^genome, and a very low homology existed between the E^e ^and Ns genomes. Southern hybridization of genomic DNA and meiotic pairing in hybrids of *Leymus *species confirmed that *Th. bessarabicum *(E^b^) or *Lo. elongatum *(E^e^) is not an ancestral genome of *Leymus *[8,57].

In this study, the Xm-type *Acc1 *sequence, which is homoeologous sequences of the Ns-type *Acc1 *sequences, were obtained from nearly all the sampled *Leymus *species. Genealogical analyses of the *Acc1 *gene indicated that the obvious Xm genome specific clade (clade I) was distinct from the Ns clade (clade II). The Xm-type sequence of *Leymus *was grouped with the sequences from *Agropyron *species and *Eremopyrum triticeum*. They had a 4-bp TATA insertion, while this insertion was absent in the Ns-type sequence of *Leymus *and other diploid species in Triticeae. Recent genealogical analysis of chloroplast *trnL-F *sequence in *Leymus *species suggested that five *Leymus *species from North America (*L. ambiguus*, *L. cinereus*, *L. innovatus*, *L. salinus *and *L. trticoides*) and two *Leymus *species from East Asia (*L. coreanus *and *L. komarovii*) were clustered with *Agropyron *and *Eremopyrum *species (Sha et al., unpublished data). The close relationship between *Agropyron *and *Eremopyrum *species have been supported by morphological analysis [58] and several molecular data [21]. Previous studies based on cytogenetics [59] and DNA sequence analyses [60] suggested the non-monophyletic origin of *Eremopyrum*. Our *Acc1 *sequence data showed that *Eremopyrum distans *is differentiated from *Er. triticeum*, and did not contradict the idea of paraphyly in *Eremopyrum*. Despite the non-monophyly of *Eremopyrum*, it is most likely that the Xm genome of *Leymus *species may be closely related to the P genome from *Agropyron *and the F genome of *Eremopyrum*. Equal numbers of fixed differences and shared polymorphisms (S_F _= 8, S_S _= 8) between the Xm-type sequence of *Leymus *and the sequences from *Agropyron *species and *Er. triticeum *were observed, which could be the result of incomplete lineage sorting of ancestral polymorphisms after speciation events. Molecular dating analyses suggested that the time to the most recent common ancestor (*t*MRCA) of *Leymus *(excluded *L. komarovii*), *Agropyron *and *Er. triticeum *was dated to 7.7-8.9 MYA, and the divergence of *Agropyron *and *Er. triticeum *was dated to 4.3-4.6 MYA, implying that the divergence between the Xm and P/F genomes might be prior to the divergence between the P and F genomes. Given the present data, it is hypothesized that the Xm genome of *Leymus *species might have been originated from an ancestral progenitor which was closely related to *Agropyron *species and *Er. triticeum*.

### Colonization of Leymus

The present results substantiate that the Ns genome of *Leymus *is originated from *Psathyrostachys *and suggest that the Xm genome of *Leymus *is closely related to the P genome of *Agropyron *and the F genome of *Eremopyrum*. In fact, *Psathyrostachys*, *Agropyron *and *Eremopyrum *are all distributed in Eurasia (Most of species distributed in central Asia), and most species of *Leymus *are distributed in central Asia. Based on the morphological and geographic data, many scholars suggested that the origin of the genus *Leymus *was in central Asia [2-4,6].

However, several *Leymus *species are found mainly in the mountains of North America [2,4,5]. Based on analysis of chloroplast *trnL-F *sequences, Sha et al. [61] suggested that the Xm genome lineages were suggested as the maternal donor of four *Leymus *species from North America and two *Leymus *species from East Asia. The present *Acc1 *gene data also indicated that the *Leymus *species from North America is closely related to those from central Asia and East Asia. The occurrence of similar maternal donor and close phylogenetic relationship between North American and East Asia *Leymus *species suggests a recent origin of North American plants. Molecular dating based on the intron data of the *Acc1 *gene suggested that the age of the most recent common ancestor (MRCA) of *Leymus *was dated to 11-12 MYA, and divergence time among East Asia, central Asia and North American species was dated to 6-8 MYA. Of interest, our estimated divergence time of 3.7-4.3 MYA (node 4 and 11) among most central Asia, East Asia and North America species was prior to the initial breakup of the Bering Land Bridge which was available for floristic exchanges until about 3.5 MYA [62]. Therefore, it can be suggested that North American *Leymus *species might originate from recent colonization via the Bering land bridge.

### Adaptive radiation of Leymus

Polyploids usually have an advantage in colonizing new habitats and ecological niches, a feature called adaptive radiation [16,63]. Adaptive radiation are often characterized by short branch lengths, little sequence variation, rapid speciation, and diverse morphology and ecological habitats [63-67]. The present exon + intron and intron tree showed several star-like topology (many short internal branches connecting long external branches) within *Leymus *species (subclade A, subclade D, and subclade E). The *Acc1 *gene sequence divergence was also generally relatively low, ranging from 0.2% in many cases to 3.3% (subclade A, 0.3%-1.9%; subclade D, 0.2%-2.0%; subclade E, 0.3%-1.3%), which resulted in low branching resolution in the phylogenetic trees. The occurrence of a star-like radiation was presented in the ML tree and MJ network of the exon data. In addition, significantly negative values of *F*s statistics and unimodal mismatch distributions within those polytomies observed in phylogenetic tree suggest rapid lineage expansions. These results, in combination with the fact that *Leymus *species exhibited large variation in morphological characteristics and habitats, indicate that adaptive radiation had occurred in *Leymus*.

Adaptive radiation might have been driven by specific geographical events and climate oscillations [64]. Geological evidence indicates that extensive habitat changes occurred in the Qinghai-Tibetan Plateau and adjacent areas due to the recent large-scale upliftings of the Qinghai-Tibetan Plateau in Pliocene and climate oscillations in the Quaternary within 5 MYA (The most notable Pliocene/Pleistocene event is known as the Qingzang Movement occurred 3.4 MYA, 2.5 MYA and 1.7 MYA, respectively) [68]. The present molecular dating suggested that *Leymus *have diverged in two extensive radiations during a period of 3.7-4.3 MYA and 1.7-2.1 MYA. Therefore, it is suggested that the radiation of *Leymus *might have been triggered by the recent uplifts of the Qinghai-Tibetan Plateau and the Quaternary climate oscillations. This hypothesis is further strengthened by the expansion of the habitats of its current species. The habitats preferred by most Eurasia *Leymus *species are cold and dry alpine meadow, steppe desert and dry slopes. However, *L. duthiei *grows in shady and moist forests in eastern part of the Qinghai-Tibetan Plateau (Hengduan Mountainous regions). Shi et al. [69] pointed out that both arid habitats at higher latitudes and warm and moist climate conditions at lower latitudes might result from the uplifting of the Qinghai-Tibetan Plateau. An et al. [70] suggested that the drier climate as well as biological and ecological diversity in central Asia also resulted from the uplifting of the Qinghai-Tibetan Plateau. Therefore, the rich geological and ecological diversity of the plateau and adjacent areas of central Asia, together with habitat isolation due to changing climatic conditions during and after the uplifts of the plateau, might well have promoted rapid speciation and radiation of *Leymus *in small, isolated populations. Wang et al. [66] considered that the rapid speciation and radiation could have resulted in small numbers of synapomorphic nucleotide substitutions and might promote the fixation of unique morphological characters in some species. In this study, the Ns-type sequences of *Leymus *species from the Qinghai-Tibetan Plateau have evolutionarily distinguished from those from other parts of central Asia, East Asia and North America. In particular, the species from the Qinghai-Tibetan Plateau shared a 33-bp insertion in the Ns-type sequences compared to *Psathyrostachys *species and other *Leymus *species. Morphologically, the *Leymus *species from the Qinghai-Tibetan Plateau are distinguished from the other *Leymus *species by leaf sheath glabrous, rachis and rachilla pubescent, and glume shorter than spikelet, while *L. duthiei *differs from the other species of subclade E by its highly reduced glumes and loosely flat leaf.

## Conclusion

In this study, two homoeologues of the single-copy nuclear *Acc1 *gene were isolated from almost all the polyploid *Leymus *species and were analyzed with those of nearly all the diploid taxa in Triticeae. Sequence diversity patterns and genealogical analyses suggested that *Leymus *is closely related to *Psathyrostachys*, *Agropyron*, and *Eremopyrum*, which could shed new light on our understanding of the origin of Xm genome, the polyploidization events and evolutionary history of *Leymus*. Molecular dating, in combination with geographic evidence and the distribution of diploid donor of *Leymus*, indicated that North America *Leymus *species might originate from colonization via the Bering land bridge. The *Acc1 *gene data analyses also suggested that adaptive radiation might have occurred in *Leymus *within a relatively short timeframe. This might have promoted rapid sympatric or allopatric speciation, and allowed the fixation of unique morphological characters in *Leymus*. Adaptive radiation might have led to the rich diversity, the numbers of species and widely ecological adaptation of *Leymus *species.

## Authors' contributions

FX designed the study, designed allele-specific primer, carried out data analyses and wrote the manuscript; SLN, YRW and KHY carried out part of experiments; ZHQ, DCB and ZL collected seed materials; ZYL gave the good suggestions in the experiments and manuscript; ZYH planned the study, participated in the design of the experiments, and revised the manuscript. All authors approved the final manuscript.

## Supplementary Material

Additional file 1 **Table S1**. The sectional delimitation of *Leymus *by different scholars Click here for file

Additional file 2 **Table S2**. *Leymus *species and other related genera in Triticeae used in this study. Click here for file

Additional file 3 F**ull Figure 1**. Partial alignment of the amplified *Acc1 *sequences from *Leymus *and its affinitive species used in this study. Click here for file

Additional file 4 **Full Figure 2**. Complete ML tree inferred from the exon + intron sequences of the *Acc1 *gene of *Leymus *and its affinitive species. Mismatch distribution and *F*s statistic for the *Acc1 *sequence of taxa at the node marked with black dot was showed in boxed subset. Click here for file

Additional file 5 **Full Figure 3**. Complete 50% majority-rule Bayesian tree inferred form the intron sequences of nuclear *Acc1 *gene of *Leymus *and its affinitive species. The estimated divergence dates for nodes labeled 1-13 was showed in the top-left table. Click here for file

Additional file 6 **Full Figure 4**. Complete ML tree (left) and MJ networks (right) based on exon haplotype of *Leymus *and its affinitive species. Click here for file
