# Supplementary material for: Phylogeny and evolutionary history of Leymus (Triticeae; Poaceae) based on a single-copy nuclear gene encoding plastid acetyl-CoA carboxylase
Source: BMC Evol Biol. 2009 Oct 8;9:247. doi: 10.1186/1471-2148-9-247 (PMC2770499; doi:10.1186/1471-2148-9-247)
Supplement: Additional file 1 — Table S1. The sectional delimitation of Leymus by different scholars [file 1471-2148-9-247-S1.PDF]

Additional File 1

Table S1 The sectional delimitation of *Leymus* by different scholars

Taxon	Tzvlev （1976）	Löve （1984）	Barkworth (1984)	Zhi and Teng (2007)	Yen et al. (2008)
L. akmolinensis (Drobow) Tzvel.	*Aphanoneuron*	*Aphanoneuron*	--	--	*Pratensus*
L. ambiguus (Vasey & Scribner) D.R. Dewey	--	--	*Anisopyrum*	--	*Pratensus*
L. angustus (Trin.) Pilger	*Aphanoneuron*	*Aphanoneuron*	*Anisopyrum*	*Leymus*	*Pratensus*
L. arenarius (L.) Hochst.	*Leymus*	*Leymus*	*Leymus*	--	*Arenicolus*
L. chinensis (Trin.) Tzvel.	*Anisopyrum*	*Anisopyrum*	--	*Leymus*	*Pratensus*
L. cinereus (Trin.) Tzvel.	--	*Anisopyrum*	*Anisopyrum*	--	*Pratensus*
L. coreanus (Honda) K.B.Jensen and R.R.–C Wang	--	--	--	--	*Silvicolus*
L. crassiusculus L.B. Cai	--	--	--	*Racemosus*	--
L. duthiei (Stapf) Y.H. Zhou et H.Q. Zhang	--	--	--	--	*Silvicolus*
L. duthiei var. *longearistatus* (Hack.) Y.H. Zhou et H.Q. Zhang	--	--	--	--	*Silvicolus*
L. flexus L.B. Cai	--	--	--	*Leymus*	--
L. innovatus (Beal) Pilger	--	*Anisopyrum*	*Anisopyrum*	--	*Pratensus*
L. karelinii (Turcz.) Tzvel.	*Aphanoneuron*	*Aphanoneuron*	--	*Leymus*	*Pratensus*
L. komarovii (Roshev.) J.L. Yang et C. Yen	--	--	--	--	*Silvicolus*
L. leptostachys L.B. Cai et X. Su	--	--	--	*Leymus*	--
L. multicaulis (Kar. & Kir.) Tzvel.	*Anisopyrum*	*Anisopyrum*	--	*Leymus*	*Pratensus*
L. ovatus (Trin.) Tzvel.	--	--	--	*Leymus*	--
L. paboanus (Claus) Pilger	*Aphanoneuron*	*Aphanoneuron*	--	*Leymus*	*Pratensus*
L. pendulus L.B. Cai	--	--	--	*Leymus*	--
L. pseudoracemosus Yen and Yang	--	--	--	*Racemosus*	*Arenicolus*
L. qinghaicus L.B. Cai	--	--	--	*Leymus*	*Pratensus*
L. racemosus (Lam.) Tzvel.	*Leymus*	*Leymus*	*Leymus*	*Racemosus*	*Arenicolus*
L. ramosus (Trin.) Tzvel.	*Anisopyrum*	*Anisopyrum*	--	*Anisopyrum*	*Pratensus*
L. salinus (M.E.Jones) A. Löve	--	*Anisopyrum*	*Anisopyrum*	--	*Pratensus*
L. secalinus (Georgi) Tzvel.	*Aphanoneuron*	*Aphanoneuron*	--	*Leymus*	*Pratensus*
L. shanxiensis G. Zhu & S.L. Chen	--	--	--	*Leymus*	--
L. tianschanicus (Drob.) Tzvel.	*Aphanoneuron*	*Aphanoneuron*	--	*Leymus*	*Pratensus*
L. triticoides (Buck.) Pilger	--	*Anisopyrum*	*Anisopyrum*	--	*Pratensus*
L. yiwuensis N.R. Cui & D.F. Cui	--	--	--	*Leymus*	--

Notes: “--” indicates that the species was not included in sectional delimitation.
