# Supplementary material for: Phylogeny and evolutionary history of Leymus (Triticeae; Poaceae) based on a single-copy nuclear gene encoding plastid acetyl-CoA carboxylase
Source: BMC Evol Biol. 2009 Oct 8;9:247. doi: 10.1186/1471-2148-9-247 (PMC2770499; doi:10.1186/1471-2148-9-247)
Supplement: Additional file 2 — Table S2. Leymus species and other related genera in Triticeae used in this study. [file 1471-2148-9-247-S2.PDF]

Table S2 *Leymus* species and other related genera in Triticeae used in this study

Taxa	Abbreviation	Accession No.	Genome (ploidy)	Origin	GenBank Accession No.
					Acc1
Aegilops L.					
Aegilops bicornis	AEBI	ND	$\mathbf{S}^{\mathbf{b}}$ (2×)	The Middle East	**AF343521**
Aegilops longissima	AELO	ND	$\mathbf{S}^{\mathbf{l}}$ (2×)	The Middle East	**AF343531**
Aegilops searsii	AESE	ND	$\mathbf{S}^{\mathbf{s}}$ (2×)	The Middle East	**AF343529**
Aegilops sharonensis	AESH	ND	$\mathbf{S}^{\mathbf{sh}}$ (2×)	The Middle East	**AF343525**
Aegilops speltoides ssp. *ligustica*	AESL	ND	**S** (2×)	The Middle East	**AF343535**
Aegilops speltoides ssp. *speltoides*	AESP	ND	**S** (2×)	The Middle East	**AF343527**
Aegilops tauschii	AETA	ND	**D** (2×)	The Middle East	**AF343496**
Agropyron Gaertn.					
Agropyron cristatum	ACRIa	PI 277352	**P** (2×)	Russian Federation	DQ453692
Agropyron cristatum	ACRIb	PI 499388	**P** (2×)	Xinjiang, China	
Agropyron monglicum	AMON	PI 499392	**P** (2×)	Inner Mongolia, China	DQ456970
Australopyrum (Tsvelev.) Á. Löve					
Australopyrum retrofractum	AURE	PI 533013	**W** (2×)	New South Wales, Australia	DQ497807
Crithopsis (Schult.) Roshev.					
Crithopsis delileana	CDEL	ND	**K** (2×)	Greece	DQ497804
Dasypyrum (L.) Candargy					
Dasypyrum villosum	DVIL	PI 251478	**V** (2×)	Turkey	DQ456971
Dasypyrum hordeaceum	DHOR	PI 516547	**V** (2×)	Morocco	DQ497802
Eremopyrum Jaub. et Spach.					
Eremopyrum distans	EDIS	TA 2229	**F** (2×)	Afghanistun	DQ453691
Eremopyrum triticeum	DTRI	Y 206	**F** (2×)	Xinjiang, China	DQ453690

Henrardia C. E. Hubb.					
Henrardia persica	HEPE	PI 401349	**O** (2×)	Turkey	GQ228396
Heteranthelium Hochst.					
Heteranthelium piliferum	HEPI	PI 401351	**Q** (2×)	Iran	DQ497808
Hordeum Linn.					
Hordeum bogdanii	HBOG	PI 531761	**H** (2×)	Xinjiang, China	DQ319185
Hordeum chilense	HCHI	PI 531781	**H** (2×)	Chile	DQ497805
Hordeum vulgare	HVUL	Betzes	**I** (2×)	ND	**AF343509**
Lophopyrum (Host) Á. Löve					
Lophopyrum elongatum	LOEL	PI 531719	$\mathbf{E^e}$ (2×)	St. Angulf, France	DQ355219
Peridictyon O. Seber, S. Frederiksen & C. Baden					
Peridictyon sanctum	PESA	H 3841	**Xp** (2×)	Greece	GQ228397
Psathyrostachys Nevski					
Psathyrostachys fragilis	PSAF	Y 882	**Ns** (2×)	Iran	FJ449595
Psathyrostachys huashanica	PSAH	ZY 3157	**Ns** (2×)	Shaanxi, China	DQ335577
Psathyrostachys juncea	PSAJ	PI 222050	**Ns** (2×)	Afghanistun	DQ335578
Psathyrostachys lanuginosa	PSAL	Y 1567	**Ns** (2×)	Xinjiang, China	GQ228398
Pseudoroegneria (Nevski) Á. Löve					
Pseudoroegneria libanotica	PSEL	PI 228392	**St** (2×)	Iran	DQ335574
Pseudoroegneria spicata	PSES	PI 232123	**St** (2×)	Washington, USA	DQ306262
Pseudoroegneria stipifornia	PSESTI	PI 440095	**St** (2×)	Yankulskaya, Russian	DQ335576
Pseudoroegneria strigosa	PSEST	PI 499637	**St** (2×)	Xinjiang, China	DQ335575
Secale L.					
Secale cereale	SECE	Imperial	**R** (2×)	ND	**AF343516**
Taeniatherum (L.) Nevski					
Taeniatherum caput-medusae	TACA	PI 220591	**Ta** (2×)	Afghanistan	DQ497803

Thinopyrum (Savul. & Rayss) Á. Löve					
Thinopyrum bessarabicum	TBES	PI 531711	$\mathbf{E^b}$ (2×)	Crimea, Ukraine	DQ355220
Triticum L.					
Triticum monococcum	TRMO	TA 2025	$\mathbf{A^m}$ (2×)	The Middle East	**AF343517**
Triticum urartu	TRUR	TA 763	**A** (2×)	Lebanon	**AF343518**
Leymus Hochst.			**NsXm**		
Leymus akmolinensis	LAKM	PI 440306	4×	Russian Federation	EU301812
Leymus ambiguus	LAMB	PI 531795	8×	Colorado, USA	FJ449626, DQ319178
Leymus angustus	LANG	PI 531797	12×	Urumqi, Xinjiang, China	DQ319179, FJ449613
Leymus arenarius	LARE	PI 272126	4×	Alma-Ata, Kazakhstan	FJ449625, DQ319177
Leymus chinensis	LCHI	PI 499515	4×	Xilinhot, Inner Mongolia, China	FJ449609, FJ449612
Leymus cinereus	LCIN	PI 469229	8×	Saskatchewan, Canada	EU301817, EU301818
Leymus coreanus	LCOR	W6 14259	4×	Primorye, Russian Federation	DQ319184
Leymus crassiusculus	LCRA	ZY 06059	4×	Guinan, Qinghai, China	FJ449596, FJ449624
Leymus duthiei	LDUT	ZY 2004	4×	Chongzhou, Sichuan, China	DQ335570, EU301816
Leymus duthiei var. *longearistatus*	LLON	ZY 2005	4×	Tokyo, Japan	DQ335571
Leymus flexus	LFLE	ZY 06044	4×	Xinghai, Qinghai, China	FJ449614, FJ449597
Leymus innovatus	LINN	PI 236818	4×	Canada	EU301820, EU301821
Leymus karelinii	LKAR	PI 598525	12×	Dafeng, Xinjiang, China	FJ449607, FJ449629
Leymus komarovii	LKOM	ZY 06001	4×	Heilongjiang, China	EU301810, EU301811
Leymus leptostachys	LLEP	ZY 06053	4×	Guinan, Qinghai, China	FJ449602, FJ449620
Leymus multicaulis	LMUL	PI 440326	4×	Dzhambul, Kazakhstan	FJ449608
Leymus ovatus	LOVA	ZY 06039	4×	Xining, Qinghai, China	FJ449615, FJ449599
Leymus paboanus	LPAB	PI 531808	8×	Estonia	FJ449611, DQ319181
Leymus pendulus	LPEN	ZY 05003	4×	Hongyuan, Sichuan, China	FJ449605, FJ449623
Leymus pseudoracemosus	LPSE	PI 531810	4×	Geermu, Qinghai, China	EU366386, EU366387

Leymus qinghaicus	LQIN	ZY 07008	4×	Hongyuan, Sichuan, China	FJ449604, FJ449622
Leymus racemosus	LRAC	PI 478832	4×	Montana,United States	EU366388, EU366389
Leymus ramosus	LRAM	PI 499653	4×	Xinjiang, China	FJ449616
Leymus salinus	LSAL	PI 531816	8x	Uath, United States	EU366390, EU366391
Leymus secalinus	LSEC	ZY 06063	4×	Gonghe, Qinghai, China	FJ449618, FJ449600
Leymus shanxiensis	LSHA	ZY 06045	4×	Xining, Qinghai, China	FJ449601, FJ449619
Leymus tianschanicus	LTIA	Y 2036	4×	Tianshan, Xinjiang, China	FJ449606, FJ449628
Leymus triticoides	LTRI	PI 516194	4×	Oregon, United States	EU301813, EU301814
Leymus yiwuensis	LYIW	ZY 06089	4×	Qinghai, China	FJ449598, FJ449617
Bromus L.					
Bromus inermis	--	PI 618974		Xinjiang, China	EU366392

The underlined GenBank accession numbers with bold represent previously published sequences from the GenBank (http://www.ncbi.nlm.nih.gov). ND: not determined.
