# Supplementary figures and images for: Phylogeny and evolutionary history of Leymus (Triticeae; Poaceae) based on a single-copy nuclear gene encoding plastid acetyl-CoA carboxylase
Source: BMC Evol Biol. 2009 Oct 8;9:247. doi: 10.1186/1471-2148-9-247 (PMC2770499; doi:10.1186/1471-2148-9-247)

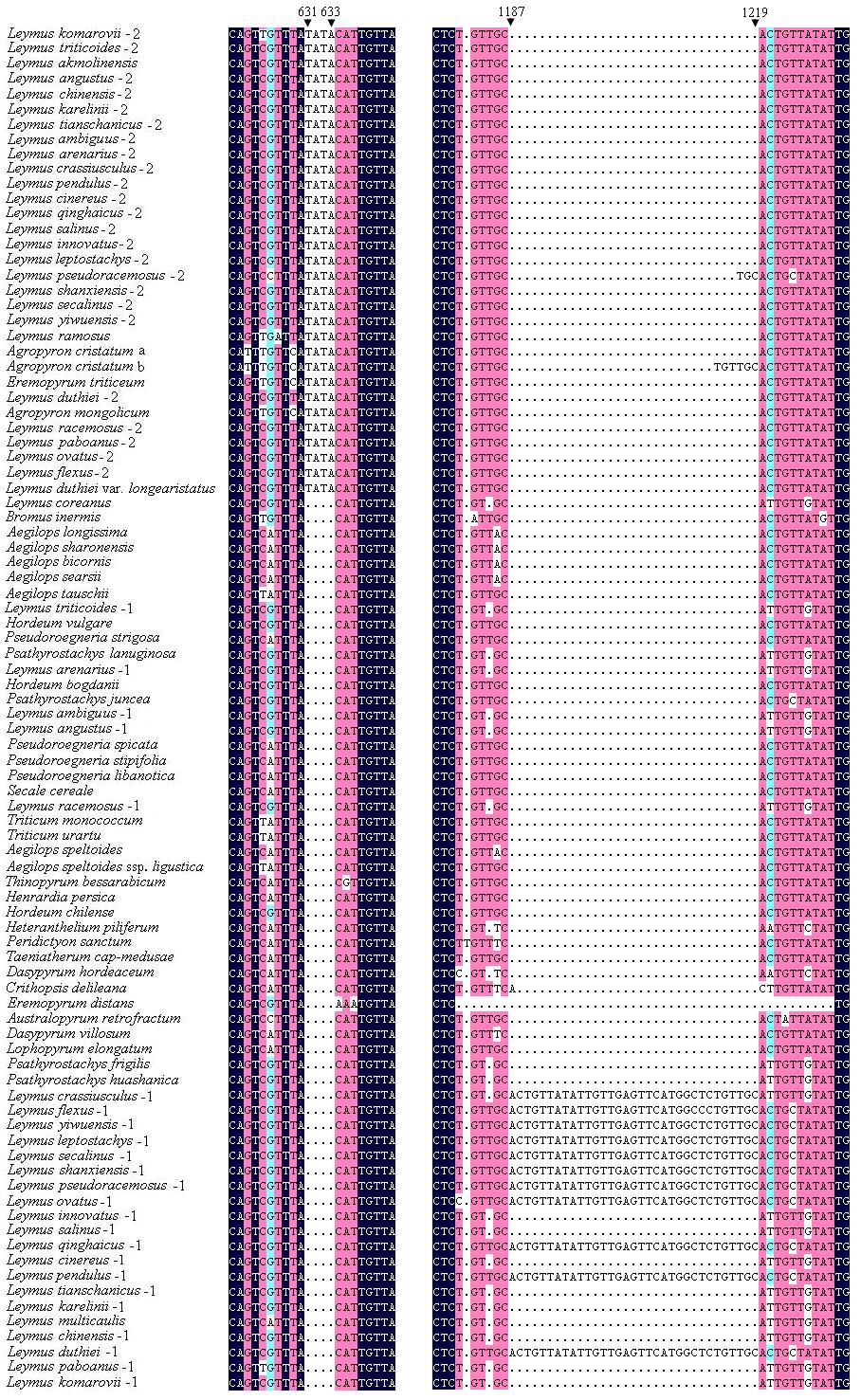

Supplement: Additional file 3 — Full Figure 1. Partial alignment of the amplified Acc1 sequences from Leymus and its affinitive species used in this study. [file 1471-2148-9-247-S3.JPEG]

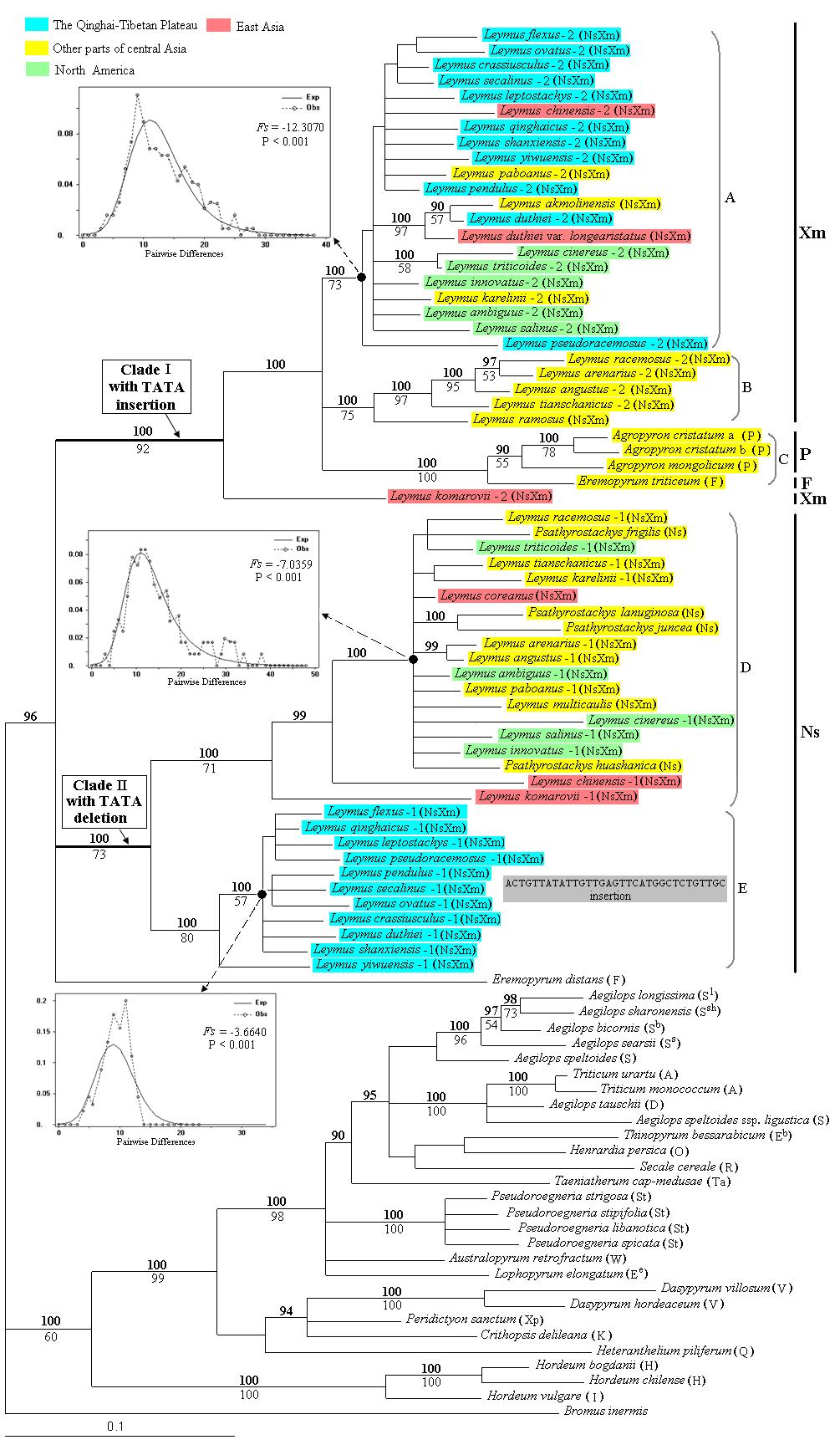

Supplement: Additional file 4 — Full Figure 2. Complete ML tree inferred from the exon + intron sequences of the Acc1 gene of Leymus and its affinitive species. Mismatch distribution and Fs statistic for the Acc1 sequence of taxa at the node marked with black dot was showed in boxed subset. [file 1471-2148-9-247-S4.JPEG]

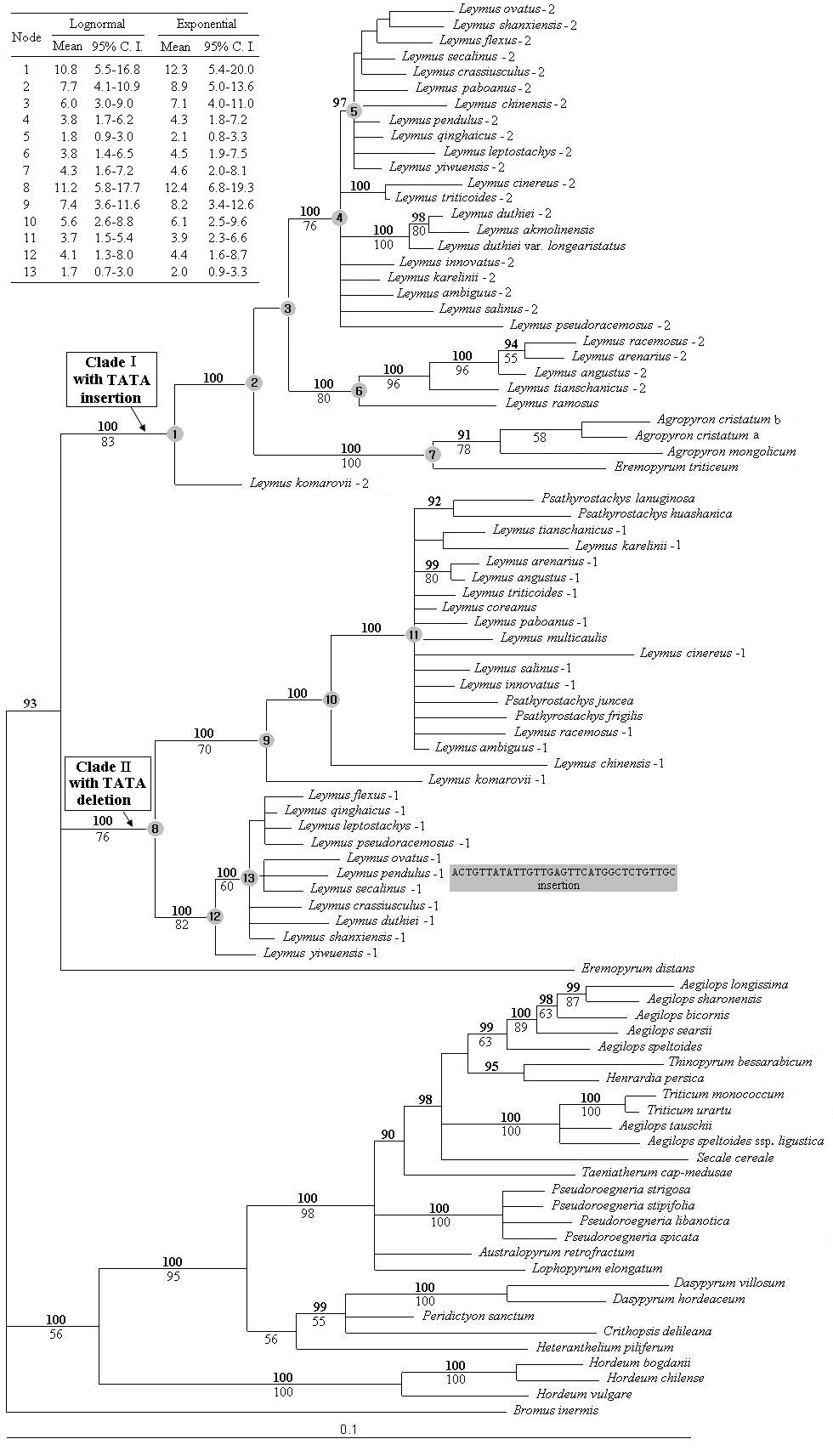

Supplement: Additional file 5 — Full Figure 3. Complete 50% majority-rule Bayesian tree inferred form the intron sequences of nuclear Acc1 gene of Leymus and its affinitive species. The estimated divergence dates for nodes labeled 1-13 was showed in the top-left table. [file 1471-2148-9-247-S5.JPEG]

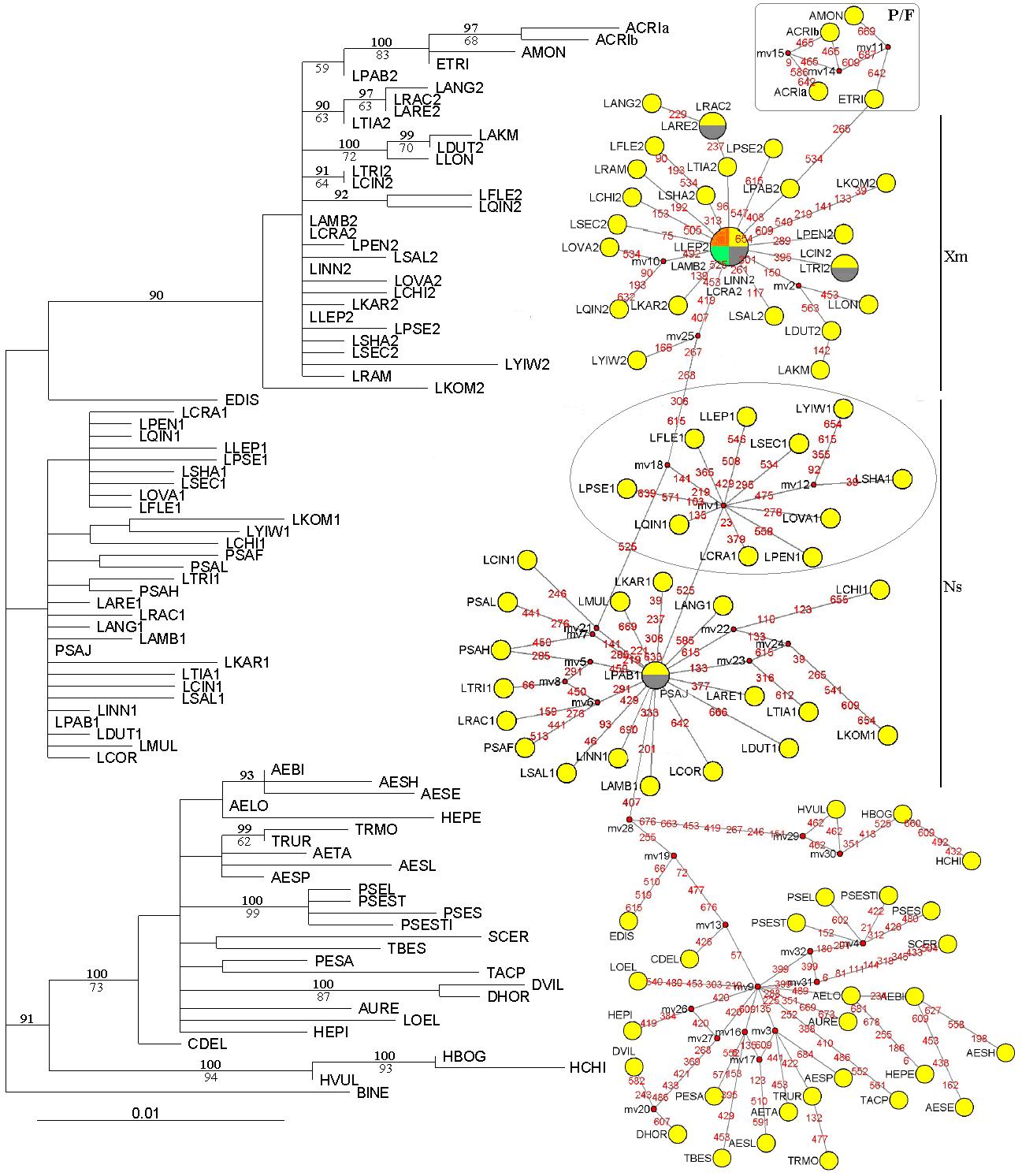

Supplement: Additional file 6 — Full Figure 4. Complete ML tree (left) and MJ networks (right) based on exon haplotype of Leymus and its affinitive species. [file 1471-2148-9-247-S6.JPEG]
